# Adhesion-Induced Phase Behavior of Two-Component Membranes and Vesicles

**DOI:** 10.3390/ijms14012203

**Published:** 2013-01-22

**Authors:** Tahereh Rouhiparkouhi, Thomas R. Weikl, Dennis E. Discher, Reinhard Lipowsky

**Affiliations:** 1 Theory & Bio-Systems, Max Planck Insitute of Colloids and Interfaces, Potsdam 14424, Germany; E-Mails: tahereh.rouhiparkouhi@mpikg.mpg.de (T.R.); thomas.weikl@mpikg.mpg.de (T.R.W.); 2 Biophysical Engineering, University of Pennsylvania, Philadelphia, PA 19104, USA; E-Mail: discher@seas.upenn.edu

**Keywords:** lipid membranes, phase separation, fluid-fluid coexistence, intramemembrane domains, membrane adhesion, phase diagrams

## Abstract

The interplay of adhesion and phase separation is studied theoretically for two-component membranes that can phase separate into two fluid phases such as liquid-ordered and liquid-disordered phases. Many adhesion geometries provide two different environments for these membranes and then partition the membranes into two segments that differ in their composition. Examples are provided by adhering vesicles, by hole- or pore-spanning membranes, and by membranes supported by chemically patterned surfaces. Generalizing a lattice model for binary mixtures to these adhesion geometries, we show that the phase behavior of the adhering membranes depends, apart from composition and temperature, on two additional parameters, the area fraction of one membrane segment and the affinity contrast between the two segments. For the generic case of non-vanishing affinity contrast, the adhering membranes undergo two distinct phase transitions and the phase diagrams in the composition/temperature plane have a generic topology that consists of two two-phase coexistence regions separated by an intermediate one-phase region. As a consequence, phase separation and domain formation is predicted to occur separately in each of the two membrane segments but not in both segments simultaneously. Furthermore, adhesion is also predicted to suppress the phase separation process for certain regions of the phase diagrams. These generic features of the adhesion-induced phase behavior are accessible to experiment.

## 1. Introduction

Multi-component membranes consisting of a small number of lipids provide simple model systems for biological membranes, which contain a huge number of different lipid and protein components. Since membranes are essentially 2-dimensional systems, they can attain different thermodynamic phases and undergo phase transitions between these phases. From a biological perspective, the most interesting phase transitions are provided by transitions between two distinct *liquid* phases, in which the membrane molecules can undergo fast lateral diffusion within all membrane domains [1]. The corresponding two-phase coexistence regions lead to critical points that belong to the universality class of the 2-dimensional Ising model [2].

The simplest examples for fluid-fluid coexistence in membranes are presumably found in binary mixtures of cholesterol and a single phospholipid. Indeed, a variety of spectroscopic methods such as deuterium nuclear magnetic resonance [3–5] were applied to such binary mixtures and provided evidence for the formation of intramembrane domains. The underlying mechanism for this domain formation has been a matter of some debate as reviewed in [6]. In this theoretical study, we will not address this controversy but take the fluid-fluid coexistence of cholesterol/phospholipid mixtures proposed in [4,5,7] and recently reviewed in [8] as a motivation to study a generic model for this kind of two-phase coexistence.

For ternary mixtures consisting of an unsaturated phospholipid, sphingomyelin, and cholesterol, as originally studied in the context of sphingolipid-cholesterol rafts [9], the formation of liquid-ordered and liquid-disordered domains can be directly observed by fluorescence microscopy. In this way, phase separation in ternary mixtures has been studied for a variety of membrane systems including giant vesicles [10–15], solid-supported membranes [16–18], hole-spanning (or black lipid) membranes [19], as well as pore-spanning membranes [20]. The phase diagrams of such three-component membranes have been determined using spectroscopic methods [5] as well as by fluorescence microscopy of giant vesicles and X-ray diffraction of membrane stacks [21–24]. Furthermore, fluid-fluid coexistence has also been found in giant plasma membrane vesicles that contain a wide assortment of lipids and proteins [25,26].

In this paper, we consider the effect of adhesion onto the phase behavior of multi-component membranes. We first emphasize that many adhesion geometries lead to a segmentation of the membranes. Examples are provided by the adhesion of vesicles, by hole- or pore-spanning membranes, and by membranes supported by chemically patterned surfaces. In all of these cases, the adhesion leads to two distinct membrane segments that experience different environments. These environments attract the different molecular components of the membrane with different affinities, *i.e.*, each environment acts to recruit certain components and to expel others. As a consequence, the compositions of the two membrane segments are different as well.

We will focus on the simplest example for fluid-fluid coexistence as provided by two-component membranes and study the adhesion effects by generalizing a generic lattice model for binary mixtures. This lattice model corresponds to a semi-grand canonical description and depends on the relative chemical potential for the two molecular species. Using this model, it is relatively easy to see that the adhesion-induced segmentation of the membranes leads, in general, to two distinct phase transitions in the two membrane segments. In order to obtain theoretical predictions that are accessible to experiments, we then consider the mole fractions in the two membrane segments and show how one can obtain the phase behavior in terms of these mole fractions. One important parameter turns out to be the affinity contrast, which describes the different molecular interactions between the two membrane segments and their environments.

We show that the fluid-fluid coexistence region as found for the two-component membrane in a uniform environment is replaced, for any nonvanishing affinity contrast, by two distinct coexistence regions, which are separated by an intermediate one-phase region. The relative sizes and positions of these different regions are shown to depend only on a relatively small number of parameters, namely temperature, mole fraction of one molecular species, area fraction of one of the membrane segments, and affinity contrast. For the generic case of a nonzero affinity contrast, our theory predicts that phase separation can only occur separately in each of the two membrane segments but not in both segments simultaneously. Furthermore, adhesion is also found to suppress the phase separation process within certain regions of the phase diagrams.

Our paper is organized as follows. First, Section 2 contains a brief review of fluid-fluid coexistence in two-component membranes and Section 3 describes the lattice binary mixture as a generic model for phase separation in two dimensions. In Section 4, we discuss several adhesion geometries such as vesicle adhesion and pore-spanning membranes, and describe how these geometries lead to a segmentation of the membranes into two different membrane segments. These segments experience distinct environments, which can be characterized by relative affinities. In Section 5, we introduce the lattice model for the adhering membranes and show that the relative affinities of the two membrane segments lead to shifts of the relative chemical potential. It is then relatively easy to conclude that the adhering membranes undergo two phase transitions. In order to obtain theoretical predictions that are accessible to experiments, we then replace in Section 6 the relative chemical potential by the mole fraction *X_a_* of the *a*-molecules and explain how one can obtain the phase diagrams as a function of *X_a_* and temperature. The results for these phase diagrams are then described in Section 7. At the end, we give a brief summary and outlook.

## 2. Liquid-Liquid Coexistence in Two-Component Membranes

At constant pressure, the phase diagrams of multi-component membranes depend on the ambient temperature *T* and on the composition of the membranes. For membranes consisting of two molecular species, say *a* and *b*, and containing *N_a_a*-molecules and *N_b_b*-molecules, the composition is described by the mole fractions

(1) $$X_{a}\equiv N_{a}/(N_{a}+N_{b})\;\;\;\text{and }\;\;X_{b}\equiv N_{b}/(N_{a}+N_{b})=1-X_{a},$$

one of which can be varied independently.

**Figure 1:**
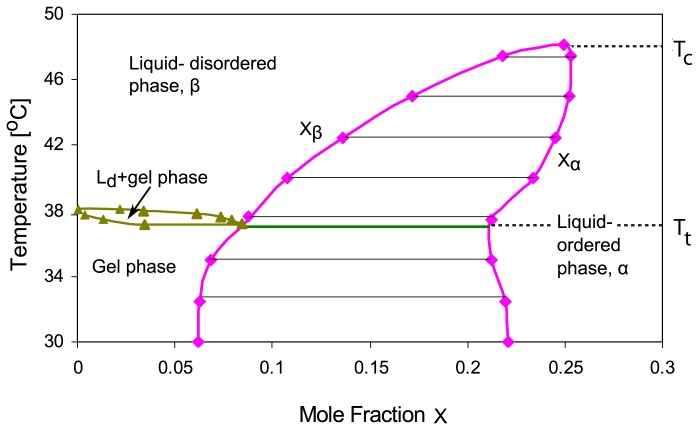
Generic phase diagram for mixed membranes consisting of cholesterol and a single phospholipid. The variable *X_a_* represents the mole fraction of cholesterol; the phase boundaries have been deduced from experiments on the phospholipid DPPC [8]. The fluid-fluid coexistence region between the liquid-ordered phase *α* on the right and the liquid-disordered phase *β* on the left is bounded by the two binodal lines *X_a_* = *X_a_*_,_*_β_*(*T*) and *X_a_* = *X_a_*_,_*_α_*(*T*), which meet at the critical point with *T* = *T_c_*. The horizontal broken lines represent tie lines within the two-phase coexistence region. The triple point temperature is denoted by *T_t_*.

Thus, phase diagrams of two-component membranes are conveniently described in the (*X_a_, T*)-plane. For binary mixtures of phospholipid and cholesterol, the phase diagrams deduced from deuterium nuclear magnetic resonance spectroscopy exhibit a coexistence region for the liquid-ordered phase, say *α*, which is rich in cholesterol, and the liquid-disordered phase, say *β*, which is poor in cholesterol [3–5,7,8], see Figure 1.

The membrane undergoes phase separation into the *α* and *β* phases within the temperature range *T_t_< T < T_c_*, *i.e.*, above the triple point temperature *T* = *T_t_* and below the critical temperature *T* = *T_c_*. The *αβ* coexistence region can then be described by

(2) $$X_{a,\beta }(T)<X_{a}<X_{a,\alpha }(T)$$

with the two binodal lines

(3) $$X_{a}=X_{a,\beta }(T)\;\;\;\text{and }\;\;X_{a}=X_{a,\beta }(T)\;\;\;\text{for }\;\;T_{t}<T<T_{c}.$$

## 3. Lattice Model for Two-Component Membranes

A relatively simple but instructive model for two-component membranes is provided by lattice binary mixtures, in which the configurations of the two molecular components are described by occupation numbers [27–31]. The two-dimensional lattice binary mixture is equivalent to the two-dimensional Ising model. Therefore, both exact results from the Ising model as well as approximation schemes such as the mean field approximation can be used to determine the phase diagrams of this lattice model.

### 3.1. Lattice Description of Large Membrane Segments

To proceed, let us first consider a large membrane segment with total area A, which is disretized into a square lattice Ω with lattice sites *i* and lattice constant $\sqrt{A}$. The total number of lattice sites will be denoted by

(4) ∣Ω∣≡A/A.

Since the discretization lattice is characterized by a single lattice constant $\sqrt{A}$, both the *a*- and the *b*-molecules are described here by the same molecular area *A*.

Within the framework of the lattice binary mixture, the molecular configurations of the two-component membrane are now described in terms of occupation numbers

(5) $$\begin{array}{llll} n_{i} & \equiv & 1 & \text{for an }a-\text{molecule on site }i\\ & \equiv & 0 & \text{for a }b-\text{molecule on site }i. \end{array}$$

The mole fraction *X_a_* of the *a*-molecules is now given by the expectation value of the occupation number *n_i_*, *i.e.*,

(6) $$X_{a}=\langle n_{i}\rangle .$$

The total number *N_a_* of *a*-molecules is given by *N_a_* = ∑*_i_n_i_* and the total number *N_b_* of *b*-molecules by

(7) $$N_{b}=|\Omega |-N_{a}=|\Omega |-\sum_{i}n_{i}.$$

Thus, for the lattice binary mixture, the total number of *a*- and *b*-molecules is fixed and given by *N_a_* + *N_b_* = |Ω|. This constraint on the two molecular numbers implies that the lattice binary mixture describes a semi-grand canonical ensemble, see further below.

### 3.2. Configurational Energy of Binary Mixture

If two neighboring sites *i* and *j* of the lattice are both occupied by *a* molecules, these molecules have the interaction energy *U_aa_*. Likewise, two neighboring *b* molecules interact via the interaction energy *U_bb_* and two neighboring sites occupied by one *a*- and one *b*-molecule contribute the interaction energy *U_ab_*. For the unbound membrane segment, the configuration-dependent interaction energy is then given by

(8) $$E_{\text{int}}\{n\}\equiv \sum_{\langle ij\rangle }\left[U_{aa}n_{i}n_{j}+U_{ab}n_{i}(1-n_{j})+U_{ab}(1-n_{i})n_{j}+U_{bb}(1-n_{i})(1-n_{j})\right]$$

and the total configurational energy ℰ{*n*} has the form

(9) $$E\{n\}=E_{\text{int}}\{n\}-\mu_{a}\sum_{i}n_{i}-\mu_{b}\sum_{i}(1-n_{i})=E_{\text{int}}\{n\}-\mu_{a}N_{a}-\mu_{b}N_{b}$$

where we have introduced two chemical potentials *μ_a_* and *μ_b_* for the *a*- and *b*-molecules. Since the molecular numbers *N_a_* and *N_b_* satisfy the constraint (7), this configurational energy is equivalent to

(10) $$E\{n\}=E_{\text{int}}\{n\}-\Delta \mu \sum_{i}n_{i}+\mu_{b}|\Omega |$$

with the relative chemical potential

(11) $$\Delta \mu \equiv \mu_{a}-\mu_{b}.$$

The term *μ_b_*|Ω| on the right hand side of (10) can be omitted because it does not depend on the occupation numbers {*n*} and, thus, cancels out from all expectation values calculated with the statistical weight exp(−ℰ{*n*}*/k*_B_*T*).

The form (10) of the configurational energy shows explicitly that the lattice binary mixture represents a semi-grand canonical ensemble [32,33], which depends on the total number |Ω|*A* of membrane molecules and on the relative chemical potential Δ*μ*. This ensemble is appropriate here because membranes have a fixed surface area and we use the simplifying assumption that both molecular species have the same molecular area. Therefore, when the system exchanges *a*- and *b*-molecules with the corresponding chemical reservoirs, we have to remove *b*-molecules when we want to insert *a*-molecules and *vice versa*.

### 3.3. Phase Behavior of Binary Mixture

The lattice binary mixture just described is equivalent to the two-dimensional Ising model if one expresses the molecular configurations {*n*} in terms of the spin variables *σ_i_* ≡ 2*n_i_* − 1. This equivalent Ising model depends only on two parameters, the dimensionless temperature

(12) $$\bar{T}\equiv \frac{k_{\text{B}}T}{J}=\frac{4k_{\text{B}}T}{2U_{ab}-U_{aa}-U_{bb}}$$

and the dimensionless ordering field

(13) $$\bar{H}\equiv \frac{H}{k_{\text{B}}T}=\frac{\frac{1}{2}\Delta \mu -(U_{aa}-U_{bb})}{k_{\text{B}}T}.$$

The Ising system undergoes phase separation for *H̄* = 0 and 0 ≤ *T̄ < T̄_c_*, where *T̄_c_* denotes the critical temperature. The exact value of the critical temperature is given by [34]

(14) $${\bar{T}}_{c}=\frac{2}{\text{ln}(1+\sqrt{2})}≃2.27$$

for the Ising model on a square lattice. As we approach the line *H̄* = 0 from positive and negative values of *H̄*, the order paramter 〈*σ_i_*〉 attains the values 〈*σ_i_*〉 = + ϒ (*T̄*) and 〈*σ_i_*〉 = −ϒ (*T̄*), respectively, with the spontaneous order parameter [34]

(15) $$\begin{array}{llll} ϒ(\bar{T}) & = & {\left[1-\text{sinh}{\left(2/\bar{T}\right)}^{-4}\right]}^{1/8} & \text{for }\bar{T}\leq {\bar{T}}_{c}\\ & = & 0 & \text{for }\bar{T}\geq {\bar{T}}_{c}. \end{array}$$

The corresponding phase transition in the lattice binary mixture occurs at the relative chemical potential

(16) $$\Delta \mu =2(U_{aa}-U_{bb})\equiv \mu_{\alpha \beta }\;\;\;\text{for }\;\;0\leq \bar{T}<{\bar{T}}_{c}.$$

As we approach the transition value Δ*μ* = *μ_αβ_* of the relative chemical potential from below or from above, the mole fraction *X_a_* = 〈*n_i_*〉 approaches the values

(17) $$X_{a,\beta }(\bar{T})=\frac{1}{2}-\frac{1}{2}ϒ(\bar{T})\;\;\;\text{and }\;\;X_{a,\beta }(\bar{T})=\frac{1}{2}+\frac{1}{2}ϒ(\bar{T}),$$

respectively, with the function ϒ (*T̄*) as given by (15). These two mole fractions define the binodal lines *X_a_* = *X_a_*_,_*_β_*(*T̄*) and *X_a_* = *X_a_*_,_*_α_* in the (*X_a_, T̄*) phase diagram of the lattice binary mixture. This phase diagram is symmetric with respect to the transformation $X_{a}\to X_{a}^{\prime }=\frac{1}{2}-X_{a}$, which reflects the particle-hole symmetry of the lattice binary mixture.

### 3.4. Relative Chemical Potential as a Function of Mole Fraction

The semi-grand canonical ensemble of the lattice binary mixture is not particularly convenient from an experimental point of view since the relative chemical potential Δ*μ* does not represent an experimental control parameter. Instead of Δ*μ*, one typically controls the mole fraction *X_a_*. If we consider *X_a_* as an independent thermodynamic variable, the relative chemical potential becomes a dependent variable that will be described by the functional relationship

(18) $$\Delta \mu =G(X_{a}).$$

The change from Δ*μ* to *X_a_* corresponds to a Legendre transformation from the semi-grand canonical ensemble to the canonical ensemble. Even though the form of the function *G* is not known explicitly (because we have no exact solution for the 2-dimensional Ising model in a finite ordering field, *H̄* ≠= 0), thermodynamics implies some useful properties of *G*(*X_a_*).

First, it follows from thermodynamic stability that the chemical potential *μ_a_* of the *a*-molecules must be a non-decreasing function of *X_a_*, see, e.g., [35]. Likewise, the chemical potential *μ_b_* of the *b*-molecules must be a non-decreasing function of *X_b_* = 1 − *X_a_*, which implies that it must be a non-increasing function of *X_a_*. It then follows that the relative chemical potential Δ*μ* = *μ_a_* − *μ_b_* as a function of *X_a_* must satisfy

(19) $$\Delta \mu =G(X_{a})\;\;\;\text{with }\;\;\partial G/\partial X_{a}\geq 0.$$

More precisely, the function Δ*μ* = *G*(*X_a_*) has a strictly positive derivative,

(20) $$\partial G/\partial X_{a}>0\;\text{within a one}-\text{phase region}$$

and stays constant with

(21) $$G(X_{a})=\mu_{\alpha \beta }\;\;\;\text{and }\;\;\partial G/\partial X_{a}=0\;\;\;\text{within a two}-\text{phase coexistence region}.$$

It then follows that *G*(*X*) is a monotonically increasing function of *X* for 0 *< X < X_a_*_,_*_β_*( *T̄*), stays constant for *X_a_*_,_*_β_*(*T̄*) ≤ *X* ≤ *X_a_*_,_*_β_*(*T̄*), and continues to increase monotonically for *X_a_*_,_*_β_*(*T̄*) *< X <* 1, where the two binodals are explicitly given by (17).

## 4. Membrane Adhesion and Segmentation

The adhesion of membranes and vesicles often leads to different membrane segments, in which the molecules experience distinct environments. Such a segmentation is found both for adhering vesicles and for solid-supported membranes. As explained in the following subsections, these adhering membranes often consist of two segments that are exposed to two different environments.

### 4.1. Adhesion of Vesicles

The adhesion of vesicles has been studied for a long time, see, e.g., [36,37] for one-component membranes and [1,38] for multi-component membranes. Here, we will focus on the strong adhesion regime, in which the vesicle attains the shape of a spherical cap. The same shape has also been observed for the strong adhesion of red blood cells [39,40].

#### 4.1.1. Strong Adhesion Regime

In general, a vesicle that sticks to a planar substrate surface exhibits two membrane segments: an unbound segment $S^{\left[1\right]}$ that is not in contact with the substrate surface and a bound segment $S^{\left[2\right]}$ that forms the vesicle’s contact area. The surface areas of the unbound and bound membrane segments will be denoted by $A^{\left[1\right]}$ and $A^{\left[2\right]}$, respectively. The overall area A of the membrane is then given by

(22) $$A=A^{\left[1\right]}+A^{\left[2\right]}.$$

The two membrane segments meet at the contact line of the vesicle. Along this line, the unbound membrane segment has the contact curvature radius [36]

(23) $$R_{\text{co}}={\left(\kappa /2|W|\right)}^{1/2},$$

which depends on the membrane’s bending rigidity *κ* and the adhesion free energy *W* per unit area. We use the convention that the free energy density *W* is *negative* for an attractive substrate surface.

The strong adhesion regime considered here corresponds to the situation, in which

(24) $$R_{\text{co}}\ll R_{\text{ve}}\equiv {\left(A/4\pi \right)}^{1/2},$$

*i.e.*, in which the contact curvature radius *R*_co_ is much smaller than the vesicle size *R*_ve_. This inequality is equivalent to

(25) $$|W{|}^{1/2}\gg {\left(2\pi \kappa /A\right)}^{1/2}$$

where the quantity κ/A may be regarded as the membrane tension arising from the closure of the vesicle membrane.

Let us now focus on giant vesicles that are conveniently studied by optical microscopy. The size *R*_ve_ of such vesicles usually exceeds 20 *μ*m. Therefore, these vesicles are in the strong adhesion regime as soon as the contact curvature radius is below optical resolution, *i.e.*, *R*_co_< L_*_≃ 0.5 *μ*m or

(26) $$|W|>|W{|}_{*}\equiv \frac{\kappa }{2L_{*}^{2}}≃\frac{\kappa }{0.5\;\mu {\text{m}}^{2}}.$$

**Figure 2:**
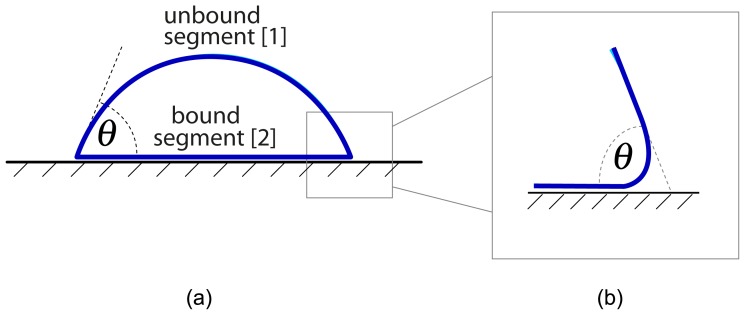
(**a**) Side view of a vesicle that strongly adheres to a planar substrate surface. The vesicle membrane (blue) consists of two segments: the unbound segment $S^{\left[1\right]}$ that forms a spherical cap with surface area $A^{\left[1\right]}$ and the bound segment $S^{\left[2\right]}$ with surface area $A^{\left[2\right]}$. The two segments meet along the contact line with effective contact angle *θ* = *θ*_eff_; and (**b**) When viewed with increased resolution, the unbound membrane segment close to the contact line is smoothly curved [36].

For a lipid bilayer, the bending rigidity *κ* is of the order of 10^−19^ J or 24 *k_B_T_o_* at room temperature *T_o_* = 25 °C, which implies |*W*|_*_ ≃ 2 × 10^−4^ mJ/m^2^ or |*W*|_*_ ≃ 0.5 *k_B_T/*(100 nm)^2^. Thus, the strong adhesion regime should typically apply as long as the adhesion is mediated by a large number of molecular interactions. In this regime, the vesicle spreads onto the surface as much as possible. For fixed vesicle volume V and fixed membrane area A, the shape with the largest contact area is provided by a spherical cap, which can be characterized by an effective contact angle *θ*_eff_ as shown in Figure 2.

#### 4.1.2. Area Fractions

The total surface area A plays the role of the basic area scale. The spherical cap shape is then determined by A and one other geometric parameter such as the effective contact angle *θ*_eff_, see Figure 2, or the reduced volume

(27) $$v\equiv \frac{3V/4\pi }{{\left(A/4\pi \right)}^{3/2}},$$

which can be controlled by osmotic deflation and inflation. Because we focus here on the adhesion-induced segmentation of the membrane, it will be convenient to choose the area fraction

(28) $$q^{\left[1\right]}\equiv \frac{A^{\left[1\right]}}{A}=\frac{A^{\left[1\right]}}{A^{\left[1\right]}+A^{\left[2\right]}}$$

of the unbound membrane segment $S^{\left[1\right]}$ as the second geometric parameter. The area fraction *q*^[2]^ of the bound segment $S^{\left[2\right]}$ is then given by *q*^[2]^ = 1 − *q*^[1]^. The relations between *q*^[1]^ and the other geometric parameters such as the reduced volume *v* are discussed in Appendix A at the end of this paper.

The area fraction *q*^[1]^ turns out to be an important parameter in order to describe the phase diagram of multi-component membranes. This parameter can vary within the range

(29) $$\frac{1}{2}\leq q^{\left[1\right]}<1\;\;\;(\text{adhering vesicle}).$$

The limiting cases correspond to a flat pancake with $q^{\left[1\right]}=\frac{1}{2}$ and to a sphere touching the surface in a single point, which implies *q*^[1]^ = 1. For a hemi-sphere, the area fraction *q*^[1]^ = 2*/*3. Because the area fraction *q*^[1]^ is uniquely determined by the reduced volume *v*, see Appendix A, it can be varied experimentally by changing the osmotic conditions leading to osmotic deflation or inflation of the vesicle.

### 4.2. Supported Membranes

A variety of methods has been developed in order to immobilize membranes on solid or rigid substrate surfaces. Here, we will discuss several support geometries that lead to two membrane segments and, thus, can again be characterized by the area fraction *q*^[1]^ of the segments $S^{\left[1\right]}$. Membranes supported by uniform substrate surfaces then correspond to the limiting cases *q*^[1]^ = 0 and *q*^[1]^ = 1.

#### 4.2.1. Partially Supported Membranes

**Figure 3:**
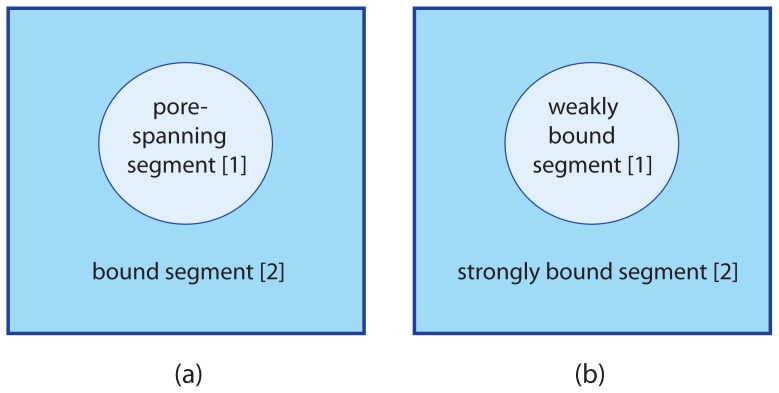
(**a**) Top view of a partially supported membrane spanning a single circular hole or pore within the rigid support. The membrane consists of a pore-spanning or unbound segment $S^{\left[1\right]}$ with area $A^{\left[1\right]}$ and a bound segment $S^{\left[2\right]}$ with area $A^{\left[2\right]}$; and (**b**) Membrane supported by a chemically patterned surface with a single, circular surface domain that attracts the membrane less strongly than the surrounding substrate. The membrane is then divided up into a weakly bound segment $S^{\left[1\right]}$ with area $A^{\left[1\right]}$ and a strongly bound segment $S^{\left[2\right]}$ with area $A^{\left[2\right]}$. If the circular surface domain does not attract or repel the membrane, the adhesion geometry in (**b**) is thermodynamically equivalent to the one in (**a**).

Two examples for partially supported membranes are provided by hole-spanning membranes, also known as black lipid membranes, see, e.g., [19], as well as by pore-spanning membranes as in [20]. Here, a “hole” corresponds to a channel through a relatively thin rigid plate whereas a “pore” refers to a groove in a relatively thick substrate. If the hole and the pore have the same cross-section, both systems lead to essentially the same adhesion geometry as shown in Figure 3a for a circular cross-section.

For these adhesion geometries, the membrane is again divided up into an unbound segment $S^{\left[1\right]}$ spanning the hole or pore and a bound segment $S^{\left[2\right]}$ in close contact with the supporting surface. In general, the area fraction *q*^[1]^ of the hole- or pore-spanning segment can now vary in the range

(30) $$0\leq q^{\left[1\right]}<1\;\;\;(\text{pore}-\text{spanning segments})$$

where the limiting case *q*^[1]^ = 0 corresponds to a membrane supported by an adhesive surface without a hole or pore. For the present geometry, the area fraction *q*^[1]^ must always be smaller than one in order to firmly attach the membrane to the substrate surface. If the membrane spans several holes or pores, it contains several unbound segments, and the area $A^{\left[1\right]}$ is now equal to the total area of all of these unbound membrane segments.

#### 4.2.2. Membranes Supported by Chemically Patterned Surfaces

Another adhesion geometry of interest are membranes adhering to a chemically patterned surface as depicted in Figure 3b. A variety of patterning techniques have been used to produce such systems [41,42]. Here, we consider substrate surfaces that contain two types of surface domains: a substrate surface “matrix” that attracts the membrane relatively strongly and embedded surface domains that attract the membrane only weakly. If the latter attraction vanishes or the domain/membrane interactions becomes repulsive, the adhesion geometry shown in Figure 3b becomes thermodynamically equivalent to the one in Figure 3a. The example for a patterned surface as shown in Figure 3b displays a surface with a single, circular domain. In general, the surface may contain several domains and these domains may have noncircular shapes.

The area fraction *q*^[1]^ of the segments $S^{\left[1\right]}$, which represent here the more weakly bound segments, now satisfies

(31) $$0\leq q^{\left[1\right]}\leq 1\;\;\;(\text{weakly bound}\;\text{segments}).$$

The two limiting cases *q*^[1]^ = 0 and *q*^[1]^ = 1 correspond to uniform substrate surfaces with strong and weak adhesion, respectively.

### 4.3. Membrane Segments Characterized by Relative Affinities

The different adhesion geometries depicted in Figures 2 and 3 all lead to a partitioning of the membranes into two segments $S^{\left[m\right]}$ with *m* = 1*,* 2. Because these segments are exposed to different environments, the two molecular components *a* and *b* experience different interactions potentials, *U_a_*^[^*^m^*^]^ and *U_b_*^[^*^m^*^]^. We use the sign convention that

(32) $$U_{a}^{\left[m\right]}<0\;\;\;\text{and }\;\;U_{b}^{\left[m\right]}<0\;\;\;\text{for attractive interaction potentials}$$

acting on the *a*- and *b*-molecules, respectively, within the segment $S^{\left[m\right]}$.

We will now characterize each membrane segment by its relative affinity

(33) $$\Delta U^{\left[m\right]}\equiv U_{a}^{\left[m\right]}-U_{b}^{\left[m\right]}.$$

If membrane segment $S^{\left[m\right]}$ represents an unbound segment not in contact with any adhesive surface, see segment $S^{\left[1\right]}$ in Figure 2 and Figure 3a, we put *U_a_*^[^*^m^*^]^ = *U_b_*^[^*^m^*^]^ ≡ 0 which implies Δ*U*^[^*^m^*^]^ = 0 as well.

Because of the sign convention (32), the sign of the relative affinity Δ*U*^[^*^m^*^]^ depends on the relative size of the interactions between *a*- and *b*-molecules in segment $S^{\left[m\right]}$ and the environment of this segment. Thus, we have

(34) $$\begin{array}{llll} \Delta U^{\left[m\right]} & < & 0 & \text{for more sticky }a-\text{molecules}\\ & > & 0 & \text{for more sticky }b-\text{molecules}, \end{array}$$

*i.e.*, if the *a*- or the *b*-molecules within segment $S^{\left[m\right]}$ are more strongly attracted by its environment.

## 5. Lattice Model for Adhering Membranes

We will now extend the lattice binary mixture as described in Section 3 to adhering membranes with two membrane segments as in Figures 2 and 3. We are then led to consider two sublattices that experience different interactions as described by the relative affinities of the two membrane segments.

### 5.1. Two Sublattices for Two Membrane Segments

Consider an adhering membrane partitioned into two segments, $S^{\left[1\right]}$ and $S^{\left[2\right]}$. The discretization of such a membrane leads to two sublattices, Ω^[1]^ and Ω^[2]^. Sublattice Ω^[1]^ for segment $S^{\left[1\right]}$ consists of

(35) $$|\Omega^{\left[1\right]}|=A^{\left[1\right]}/A$$

lattice sites whereas sublattice Ω^[2]^ for segment $S^{\left[2\right]}$ has

(36) $$|\Omega^{\left[2\right]}|=A^{\left[2\right]}/A$$

such sites. As before, the symbol *A* denotes the area per lattice site and corresponds to the molecular area of the *a*- and *b*-molecules. The area fraction *q*^[1]^ of segment $S^{\left[1\right]}$ is now equal to

(37) $$q^{\left[1\right]}=\frac{|\Omega^{\left[1\right]}|}{|\Omega^{\left[1\right]}|+|\Omega^{\left[2\right]}|}.$$

### 5.2. Configurational Energy of Adhering Membranes

The configurations of *a*- and *b*-molecules within an adhering membrane are again described in terms of the occupation numbers {*n_i_*}, where *n_i_* = 1 represents an *a*-molecule as before. The configurational energy of this membrane now contains additional terms arising from the interaction potentials *U_a_*^[^*^m^*^]^ and *U_b_*^[^*^m^*^]^ for the *a*- and *b*-molecules with the corresponding environments. More precisely, membrane segment $S^{\left[m\right]}$ contributes the additional energy term

(38) $$E_{U}^{\left[m\right]}\{n\}=\sum_{i\in \Omega^{\left[m\right]}}\left[U_{a}^{\left[m\right]}\;n_{i}+U_{b}^{\left[m\right]}\;(1-n_{i})\right]=\sum_{i\in \Omega^{\left[m\right]}}\Delta U^{\left[m\right]}n_{i}+U_{b}|\Omega^{\left[m\right]}|$$

with the relative affinity Δ*U*^[^*^m^*^]^ as in (33) where the last *n*-independent term on the right hand side can again be omitted because it does not affect the statistical properties of the system. Adding the *U*-dependent energy term ℰ*_U_*^[^*^m^*^]^ in (38) to the standard form (10) for the configurational energy of the lattice binary mixture, the configurational energy of segment $S^{\left[m\right]}$ becomes

(39) $$E^{\left[m\right]}\{n\}=E_{\text{int}}^{\left[m\right]}\{n\}+[\Delta U^{\left[m\right]}-\Delta \mu ]\sum_{i\in \Omega^{\left[m\right]}}n_{i}$$

where the interaction energy ℰ_int_^[^*^m^*^]^ describes the molecular interactions between the *a*- and *b*-molecules within segment $S^{\left[m\right]}$. This interaction energy now has the form

(40) $$E_{\text{int}}^{\left[m\right]}\{n\}=\sum_{\langle ij\rangle \in \Omega^{\left[m\right]}}\left[U_{aa}\;n_{i}n_{j}+U_{ab}\;n_{i}(1-n_{j})+U_{ab}(1-n_{i})n_{j}+U_{bb}(1-n_{i})(1-n_{j})\right]$$

which is identical with the expression (8) apart from the summation that now includes only nearest neighbors 〈*ij*〉 within the sublattice Ω^[^*^m^*^]^ corresponding to segment $S^{\left[m\right]}$.

The configurational energy of an adhering membrane consisting of two membrane segments is then given by

(41) $$E\{n\}=E^{\left[1\right]}\{n\}+E^{\left[2\right]}\{n\}+E^{\text{db}}\{n\}$$

where the additional energy term ℰ^db^{*n*} arises from the domain boundaries between the two membrane segments. The latter term can be ignored compared to the first two terms in (41) when we consider the limit of large membrane segments. Indeed, in this limit, the free energies obtained from the first two terms ℰ^[1]^{*n*} and ℰ^[2]^{*n*} increase as the segment areas $A^{\left[1\right]}\sim |\Omega^{\left[1\right]}|$ and $A^{\left[2\right]}\sim |\Omega^{\left[2\right]}|$ whereas the free energy arising from the domain boundary term ℰ^db^{*n*} increases only as the length of the domain boundary, which is of the order of $\sqrt{\text{min}(|\Omega^{\left[1\right]}|,|\Omega^{\left[2\right]}|)}$.

Therefore, in this large membrane limit, we are left with two membrane segments, $S^{\left[1\right]}$ and $S^{\left[2\right]}$, each of which is governed by a configurational energy of the form (39). In the semi-grand canonical ensemble of the lattice binary mixture, these large segments are only coupled via the relative chemical potential Δ*μ*, *i.e.*, via the particle reservoirs for *a*- and *b*-molecules.

### 5.3. Phase Transitions in Membrane Segments

The configurational energy (10) for the standard lattice binary mixture leads to a phase transition at the relative chemical potential Δ*μ* = *μ_αβ_* = 2(*U_aa_* − *U_bb_*) for 0 ≤ *T̄ < T̄_c_* as described by (16). Comparison of the configurational energy (39) for the membrane segment $S^{\left[m\right]}$ with the configurational energy (10) for the standard model then shows that this segment undergoes a phase transition at the relative chemical potential

(42) $$\Delta \mu =\mu_{\alpha \beta }+\Delta U^{\left[m\right]}\equiv \mu_{\alpha \beta }^{\left[m\right]}\;\;\;\text{for }\;\;0\leq \bar{T}<{\bar{T}}_{c}.$$

As long as the relative affinities Δ*U*^[^*^m^*^]^ of the two membrane segments are different, *i.e.*, as long as Δ*U*^[2]^ ≠= Δ*U*^[1]^, the two critical values *μ_αβ_*^[2]^ and *μ_αβ_*^[1]^ are different as well and the two segments undergo two distinct phase transitions. Therefore, the adhesion-induced partitioning into two membrane segments leads to two distinct phase transitions for Δ*U*^[2]^ ≠= Δ*U*^[1]^.

## 6. Phase Behavior in Terms of Mole Fractions

In order to make theoretical predictions that are accessible to experiment, we will now describe the phase behavior of the two membrane segments in terms of their mole fractions. Since the two membrane segments experience different environments and, thus, different relative affinities, they will, in general, differ in their compositions. Therefore, we have to distinguish the mole fraction *X_a_*^[1]^ in segment $S^{\left[1\right]}$ from the mole fraction *X_a_*^[2]^ in segment $S^{\left[2\right]}$. To determine the two mole fractions *X_a_*^[1]^ and *X_a_*^[2]^, we need two independent relations between these two variables. As shown in the following section, one relation is obtained from the partitioning of the total number of *a*- and *b*-molecules between the two membrane segments, the other from the chemical equilibrium between these segments.

### 6.1. Partitioning of Membrane Molecules

One relation between the two mole fractions *X_a_*^[1]^ and *X_a_*^[2]^ is provided by the partitioning of the molecules between the two membrane segments. Because the total number |Ω| = |Ω^[1]^| + |Ω^[2]^| of molecules is fixed within the adhering membrane, the mole fractions *X_a_*^[1]^ and *X_a_*^[2]^ satisfy the relation

(43) $$\frac{|\Omega^{\left[1\right]}|}{|\Omega^{\left[1\right]}|+|\Omega^{\left[2\right]}|}X_{a}^{\left[1\right]}+\frac{|\Omega^{\left[2\right]}|}{|\Omega^{\left[1\right]}|+|\Omega^{\left[2\right]}|}X_{a}^{\left[2\right]}=X_{a}$$

where *X_a_* is the overall mole fraction of the *a*-molecules as before. Using the expression (37) for the area fraction *q*^[1]^ and *q*^[2]^ = 1 − *q*^[1]^, the relation (43) becomes

(44) $$q^{\left[1\right]}X_{a}^{\left[1\right]}+(1-q^{\left[1\right]})X_{a}^{\left[2\right]}=X_{a}.$$

Note that this partitioning relation depends only on two parameters, the area fraction *q*^[1]^ of segment $S^{\left[1\right]}$ and the overall mole fraction *X_a_*.

### 6.2. Relative Chemical Potentials of Membrane Segments

Comparison of the configurational energy (39) of membrane segment $S^{\left[m\right]}$ with the configurational energy (10) of the standard lattice binary mixture shows that the relative chemical potential Δ*μ* of the standard model is replaced byΔ*μ*−Δ*U*^[^*^m^*^]^ for segment $S^{\left[m\right]}$. It then follows from (19) thatΔ*μ*−Δ*U*^[^*^m^*^]^ = *G*(*X_a_*^[^*^m^*^]^), *i.e.*, membrane segment $S^{\left[m\right]}$ is governed by the relative chemical potential

(45) $$\Delta \mu =G(X_{a}^{\left[m\right]})+\Delta U^{\left[m\right]}\equiv \Delta \mu^{\left[m\right]}(X_{a}^{\left[m\right]}).$$

As explained in Section 3.4, thermodynamic stability implies that the function *G*(*X_a_*^[^*^m^*^]^) is monotonically increasing for 0 *< X_a_*^[^*^m^*^]^*< X_a_*_,_*_β_*(*T̄*), attains the constant value

(46) $$G(X_{a}^{\left[m\right]})=\mu_{\alpha \beta }\;\;\;\text{for }\;\;X_{a,\beta }(\bar{T})\leq X_{a}^{\left[m\right]}\leq X_{a,\beta }(\bar{T})$$

and continues to increase monotonically for *X_a_*_,_*_α_*(*T̄*) *< X_a_*^[^*^m^*^]^*<* 1, where the two binodals *X_a_*_,_*_β_*( *T̄*) and *X_a_*_,_*_α_*( *T̄*) are explicitly given by (17).

### 6.3. Chemical Equilibrium Between Membrane Segments and Affinity Contrast

Since the two membrane segments can exchange *a*- and *b*-molecules by lateral diffusion, they will reach a state of chemical equilibrium with

(47) $$\Delta \mu^{\left[1\right]}(X_{a}^{\left[1\right]})=G(X_{a}^{\left[1\right]})+\Delta U^{\left[1\right]}=\Delta \mu^{\left[2\right]}(X_{a}^{\left[2\right]})=G(X_{a}^{\left[2\right]})+\Delta U^{\left[2\right]}$$

or

(48) $$G(X_{a}^{\left[1\right]})=G(X_{a}^{\left[2\right]})+\Delta U^{2,1}$$

where we introduced the *affinity contrast*

(49) $$\Delta U^{2,1}\equiv \Delta U^{\left[2\right]}-\Delta U^{\left[1\right]}=U_{a}^{\left[2\right]}-U_{b}^{\left[2\right]}-\left(U_{a}^{\left[1\right]}-U_{b}^{\left[1\right]}\right)$$

between the two membrane segments with Δ*U*^1,2^ = −Δ*U*^2,1^. Thus, chemical equilibrium as described by (48) provides a second relation between the two mole fractions *X_a_*^[1]^ and *X_a_*^[2]^. This relation becomes particularly useful if one of the two segments undergoes phase separation because we can then replace one of the *G*-terms by the constant value *μ_αβ_*. Furthermore, it is convenient to define the shifted function

(50) $$\Delta G(x)\equiv G(x)-\mu_{\alpha \beta },$$

which attains the value

(51) $$\Delta G(X)=0\;\;\;\text{for }\;\;X_{\beta }(\bar{T})\leq X\leq X_{\alpha }(\bar{T})$$

and increases monotonically with *X* outside of this interval. In terms of the shifted function Δ*G*, the chemical equilibrium relation (48) becomes

(52) $$\Delta G(X_{a}^{\left[1\right]})=\Delta G(X_{a}^{\left[2\right]})+\Delta U^{2,1}.$$

It is not difficult to see that any two mole fractions *X_a_*^[1]^ and *X_a_*^[2]^ that satify this equation and, thus, represent a solution of it do not depend on the shift *μ_αβ_* but only on the affinity contrast Δ*U*^2,1^. Indeed, we can add any constant to the functions Δ*G*(*X*) or *G*(*X*) without changing the solution to (52) or (48).

### 6.4. Phase Separation in Segment $S^{\left[1\right]}$

If segment $S^{\left[1\right]}$ undergoes phase separation, the mole fraction *X_a_*^[1]^ must have a value within the range

(53) $$X_{a,\beta }(\bar{T})\leq X_{a}^{\left[1\right]}\leq X_{a,\alpha }(\bar{T})$$

and Δ*G*(*X_a_*^[1]^) = 0 as in (51). The chemical equilibrium relation (52) then simplifies and becomes

(54) $$\Delta G(X_{a}^{\left[2\right]})=-\Delta U^{2,1}.$$

We now have to distinguish two cases corresponding to zero and nonzero affinity contrast Δ*U*^2,1^. If Δ*U*^2,1^ = 0, the molecules experience the same relative affinities in both membrane segments and thus have no preference for either segment. We then conclude that the whole membrane undergoes phase separation and that *X_a_*^[2]^ = *X_a_*^[1]^ = *X_a_*, where the second equality follows from the partitioning relation (44).

On the other hand, if Δ*U*^2,1^ ≠ 0, the chemical equilibrium relation (54) implies that Δ*G*(*X_a_*^[2]^) ≠ 0, *i.e.*, that segment $S^{\left[2\right]}$ does not undergo phase separation but represents a *spectator phase with uniform composition*. The corresponding mole fraction *X_a_*^[2]^ = *X_a_*_,*_^[2]^ satisfies the implicit equation

(55) $$\Delta G(X_{a,*}^{\left[2\right]})=-\Delta U^{2,1}=\Delta U^{1,2}$$

and stays constant as long as the mole fraction *X_a_*^[1]^ lies within the coexistence region (53) of segment $S^{\left[1\right]}$. Note the subscript * that is used, here and below, to indicate the mole fractions of a spectator phase.

Because Δ*G*(*x*) increases monotonically with *x* both for 0 *< x < X_β_* and for *X_α_< x <* 1, the implicit equation (55) implies that the mole fraction *X_a_*_,*_^[2]^ of the uniform spectator phase in segment $S^{\left[2\right]}$ *decreases monotonically* with increasing affinity contrast Δ*U*^2,1^ and satisfies

(56) $$0<X_{a,*}^{\left[2\right]}<X_{a,\beta }(\bar{T})\;\;\;\text{for }\;\;\Delta U^{2,1}>0$$

as well as

(57) $$X_{a,\alpha }(\bar{T})<X_{a,*}^{\left[2\right]}<1\;\;\;\text{for }\;\;\Delta U^{2,1}<0.$$

Furthermore, this mole fraction exhibits the limiting behavior

(58) $$\begin{array}{llll} X_{a,*}^{\left[2\right]} & \approx & 1 & \text{for large negative }\Delta U^{2,1}\\ & \approx & X_{a,\alpha }(\bar{T}) & \text{for small negative }\Delta U^{2,1} \end{array}$$

as well as

(59) $$\begin{array}{llll} X_{a,*}^{\left[2\right]} & \approx & X_{a,\beta }(\bar{T}) & \text{for small positive }\Delta U^{2,1}\\ & \approx & 0 & \text{for large positive }\Delta U^{2,1}. \end{array}$$

Therefore, as one increases the affinity contrast Δ*U*^2,1^ from large negative to small negative values, the mole fraction *X_a_*_,*_^[2]^ of the spectator phase in segment $S^{\left[2\right]}$ decreases monotonically as a function of Δ*U*^2,1^, from *X_a_*_,*_^[2]^ = 1 to *X_a_*_,_*_α_*(*T̄*) corresponding to the upper binodal. At Δ*U*^2,1^ = 0, this mole fraction jumps from the value *X_a_*_,_*_α_*(*T̄*) for the upper binodal to the value *X_a_*_,_*_β_*(*T̄*) for the lower binodal. Finally, as one increases the affinity contrast Δ*U*^2,1^ from small positive to large positive values, the mole fraction *X_a_*_,*_^[2]^ decreases monotonically from the value *X_a_*_,_*_β_*(*T̄*) to *X_a_*_,*_^[2]^ = 0.

### 6.5. Phase Separation in Segment $S^{\left[2\right]}$

Now, consider the situation, in which segment $S^{\left[2\right]}$ of the adhering membrane undergoes phase separation. Repeating the arguments of the previous subsection, it then follows that the mole fraction *X_a_*_,*_^[2]^ of segment $S^{\left[2\right]}$ satisfies

(60) $$X_{a,\beta }(\bar{T})\leq X_{a}^{\left[2\right]}\leq X_{a,\alpha }(\bar{T})$$

and that Δ*G*(*X_a_*^[2]^) = 0 as in (51). The mole fraction *X_a_*^[1]^ in segment $S^{\left[1\right]}$ then satisfies the implicit equation

(61) $$\Delta G(X_{a}^{\left[1\right]})=+\Delta U^{2,1}=-\Delta U^{1,2}$$

which implies

(62) $$\begin{array}{lllll} X_{a}^{\left[1\right]} & = & X_{a}^{\left[2\right]} & =\;X_{a} & \text{for }\Delta U^{2,1}=0\\ & = & X_{a,*}^{\left[1\right]} & \neq X_{a}^{\left[2\right]} & \text{for }\Delta U^{2,1}\neq 0 \end{array}$$

where *X_a_*_,*_^[1]^ denotes the mole fraction of the uniform spectator phase in segment $S^{\left[1\right]}$.

Using again the general properties of the function Δ*G*(*x*), we find from (61) that the mole fraction *X_a_*^[1]^ = *X_a_*_,*_^[1]^*increases monotonically* with increasing affinity contrast *U*^2,1^. More precisely, as one increases Δ*U*^2,1^ from large to small negative values, the mole fraction *X_a_*_,*_^[1]^ increases from *X_a_*_,*_^[1]^ = 0 to *X_a_*_,_*_β_*(*T̄*), jumps at Δ*U*^2,1^ = 0 from *X_a_*_,_*_β_*(*T̄*) to *X_a_*_,_*_α_*(*T̄*), and finally increases monotonically from *X_a_*_,_*_α_*(*T̄*) to *X_a_*_,*_^[1]^ = 1 as the affinity contrast Δ*U*^2,1^ is increased from small to large positive values. Thus, the mole fraction *X_a_*_,*_^[1]^ satisfies

(63) $$0<X_{a,*}^{\left[1\right]}<X_{a,\beta }(\bar{T})\;\;\;\text{for }\;\;\Delta U^{2,1}<0$$

and

(64) $$X_{a,\alpha }(\bar{T})<X_{a,*}^{\left[1\right]}<1\;\;\;\text{for }\;\;\Delta U^{2,1}>0.$$

Furthermore, this mole fraction exhibits the limiting behavior

(65) $$\begin{array}{llll} X_{a,*}^{\left[1\right]} & \approx & 0 & \text{for large negative }\Delta U^{2,1}\\ & \approx & X_{a,\beta }(\bar{T}) & \text{for small negative }\Delta U^{2,1} \end{array}$$

as well as

(66) $$\begin{array}{llll} X_{a,*}^{\left[1\right]} & \approx & X_{a,\alpha }(\bar{T}) & \text{for small positive }\Delta U^{2,1}\\ & \approx & 1 & \text{for large positive }\Delta U^{2,1}. \end{array}$$

## 7. Phase Diagrams for Adhering Membranes

From an experimental point of view, it is most useful to describe the phase behavior in terms of overall composition and temperature. The corresponding phase diagrams can be derived by combining the partitioning relation (44) with the chemical equilibrium relations as discussed in the previous subsections. Thus, inserting (i) the inequalities (53) for the mole fraction *X_a_*^[1]^ and (ii) the equalities (56)–(59) for the mole fraction *X_a_*^[2]^ = *X_a_*_,*_^[2]^ of the uniform spectator phase into the partitioning relation (44), we obtain the binodals for the two-phase coexistence region of segment $S^{\left[1\right]}$ in the (*X_a_, T̄*)-plane. Likewise, the binodals for the coexistence region of segment $S^{\left[2\right]}$ in the (*X_a_, T̄*)-plane is obtained by inserting the inequalities (60) for the mole fraction *X_a_*^[2]^ and the equalities (63)–(66) for the spectator phase mole fraction *X_a_*^[1]^ = *X_a_*^[1],*^ into the partitioning relation (44).

The two coexistence regions for the two membrane segments are separated by an intermediate one-phase region as long as the affinity contrast Δ*U*^2,1^ does not vanish, *i.e.*, as long as the two membrane segments are characterized by different relative affinities Δ*U*^[^*^m^*^]^ = *U_a_*^[^*^m^*^]^ − *U_b_*^[^*^m^*^]^. Thus, for Δ*U*^2,1^ ≠ 0 or relative affinities Δ*U*^[2]^ ≠ Δ*U*^[1]^, the adhesion-induced partitioning into two segments leads to two distinct two-phase coexistence regions within the (*X_a_, T̄*)-plane.

### 7.1. Parameter Dependence of Phase Diagrams

The phase diagrams of the adhering membranes in the (*X_a_, T̄*)-plane depend on the parameters that enter the partitioning relation (44) and the chemical equilibrium relations. The partitioning relation (44) contains only one parameter, the area fraction *q*^[1]^ of segment $S^{\left[1\right]}$, in addition to the overall mole fraction *X_a_* of the *a*-molecules. The chemical equilibrium relations, on the other hand, involves the function Δ*G*(*X*) and the affinity contrast Δ*U*^2,1^. The function Δ*G*(*X*) depends on the interaction parameters *U_aa_, U_ab_*, and *U_bb_* in the configurational energy (40) via the dimensionless temperature *T̄* ≡ 4*k*_B_*T=*(2*U_ab_* − *U_aa_* − *U_bb_*) as introduced in (12).

Therefore, for the lattice model considered here, the phase diagrams of the adhering membranes depend only on four parameters: (i) overall mole fraction *X_a_*, (ii) dimensionless temperature *T̄*, (iii) area fraction *q*^[1]^, a purely geometric parameter, and (iv) affinity contrat Δ*U*^2,1^.

**Figure 4:**
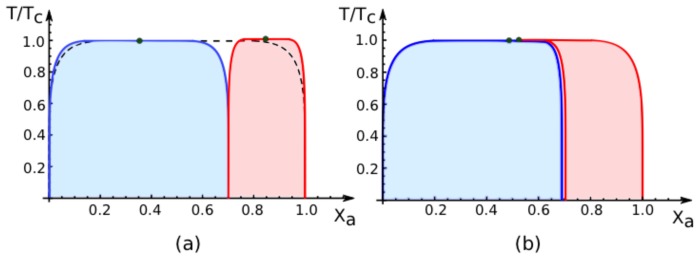
Exact phase diagrams of the lattice model for adhering membranes as a function of mole fraction *X_a_* and reduced temperature *T/T_c_* for (**a**) large and (**b**) small *positive* values of the affinity contrast Δ*U*^2,1^ = Δ*U*^[2]^ − Δ*U*^[1]^ and fixed area fraction *q*^[1]^ = 0.7 of membrane segment $S^{\left[1\right]}$. The blue and red regions represent the two-phase coexistence regions of segment $S^{\left[1\right]}$ and $S^{\left[2\right]}$, respectively. Likewise, the blue and red curves correspond to the binodal lines for these two segments. Positive values of Δ*U*^2,1^ imply that the *a*-molecules prefer to stay in segment $S^{\left[1\right]}$ corresponding to the unbound segments in Figure 2 and Figure 3a as well as to the weakly bound segment in Figure 3b. Therefore, as one increases the mole fraction *X_a_* starting from *X_a_* = 0, one first enters the blue coexistence region for segment $S^{\left[1\right]}$ and subsequently the red coexistence region for segment $S^{\left[2\right]}$. The two critical points depicted in (**b**) merge in the limit of vanishing affinity contrast, Δ*U*^2,1^ = 0. The broken line in (**a**) represents the binodals for the coexistence region of the uni-env membrane, *i.e.*, for the same two-component membrane in a uniform environment; in (**b**), the broken line is masked by the blue and red binodals. For *T >* 0, the blue and red coexistence regions in (**a**) and (**b**) are separated by an intermediate one-phase region, in which the adhering membrane does not undergo phase separation even though the uni-env membrane does.

**Figure 5:**
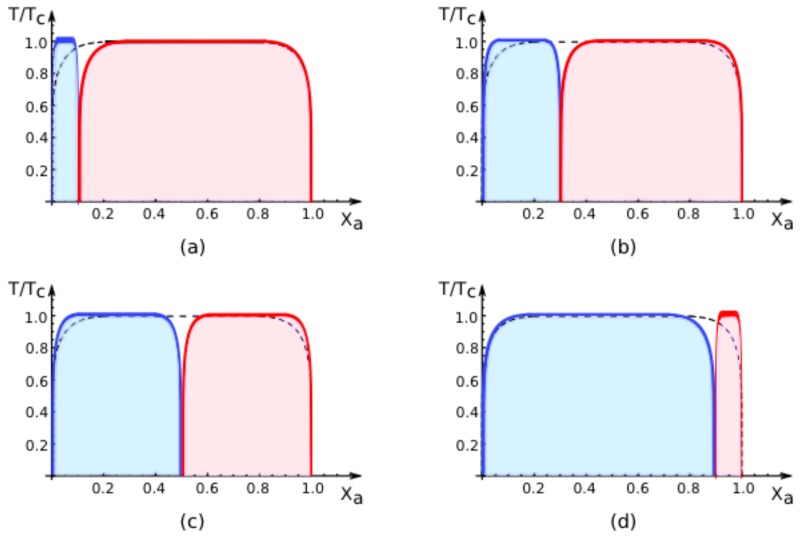
Exact phase diagrams of the lattice model for adhering membranes as a function of mole fraction *X_a_* and reduced temperature *T/T_c_* for different area fractions *q*^[1]^ = 0.1*,* 0.3*,* 0.5, and 0.9 in (**a**), (**b**), (**c**), and (**d**), respectively. The affinity contrast Δ*U*^2,1^ is kept fixed at a large positive value as in Figure 4(a). As one varies the parameter *q*^[1]^ from *q*^[1]^ = 0 up to *q*^[1]^ = 1, the coexistence regions smoothly evolve from a single red region for segment $S^{\left[2\right]}$ to a single blue region for segment $S^{\left[1\right]}$. As in Figure 4, the broken line represents the binodals of the coexistence region for the uni-env membrane, *i.e.*, for the same two-component membrane in a uniform environment.

**Figure 6:**
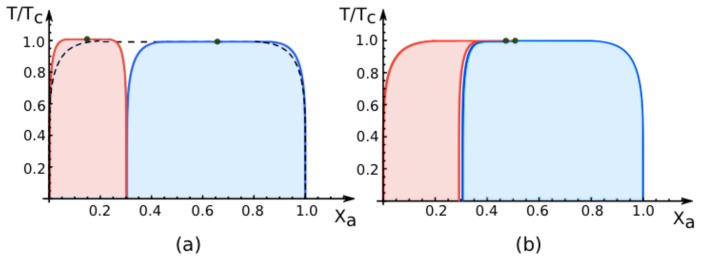
Exact phase diagrams of the lattice model for adhering membranes as a function of area fraction *X_a_* and reduced temperature *T/T_c_* for (**a**) large and (**b**) small *negative* values of the affinity contrast Δ*U*^2,1^ and fixed area fraction *q*^[1]^ = 0.7. Negative values of Δ*U*^2,1^ imply that the *a*-molecules prefer to stay in membrane segment $S^{\left[2\right]}$, corresponding to the bound segments in Figure 2 and Figure 3a as well as to the strongly bound segment in Figure 3b. The blue and red regions again represent the two-phase coexistence regions of the segments $S^{\left[1\right]}$ and $S^{\left[2\right]}$, respectively, but, compared to Figures 4 and 5, these two coexistence regions have now swapped their relative positions.

**Figure 7:**
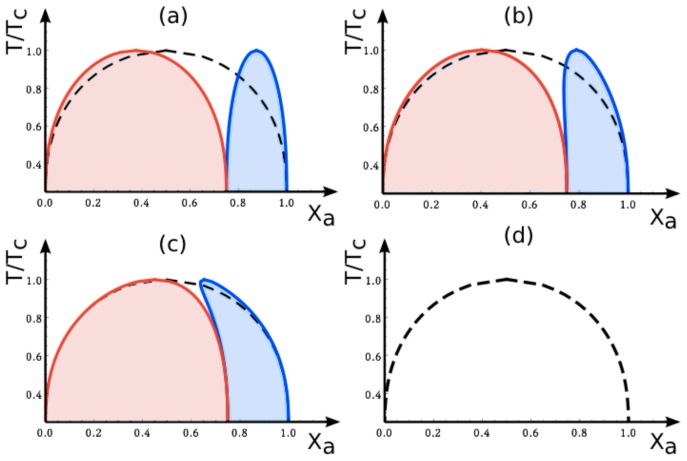
Mean-field phase diagrams of the lattice model for adhering membranes as a function of mole fraction *X_a_* and reduced temperature *T/T_c_* for fixed area fraction *q* = 0.25. The different subfigures correspond to different *negative* values of the reduced affinity contrast *Ū*^2,1^ ≡ *Ū*^2,1^*=*(*k_B_T_c_*): (**a**) *Ū*^2,1^ = −5, (**b**) *Ū*^2,1^ = −0.5, (**c**) *Ū*^2,1^ = −0.05, and (d) *Ū*^2,1^ = 0. Inspection of these subfigures shows that the blue and red coexistence regions for the two membrane segments change smoothly as the affinity contrast is increased from large negative to small negative values. Furthermore, in the limit of vanishing affinity contrast, the two coexistence regions merge into the single region for the uni-env membrane as described by the broken black lines in (**a**–**d**).

The four-dimensional parameter space is most easily explored via two-dimensional slices. In the following, we will display two-dimensional phase diagrams that depend on the overall mole fraction *X_a_* and on the rescaled temperature *T̄ = T̄_c_* = *T/T_c_* for fixed values of the area fraction *q*^[1]^ and of the affinity contrast Δ*U*^2,1^, see Figures 4, 5, 6 and 7 below. This choice is convenient because it allows a direct comparison with the phase diagram of the two-component membrane in a uniform environment. The latter phase diagram, which depends only on mole fraction *X_a_* and on temperature, is recovered for the limiting values *q*^[1]^ = 0 and *q*^[1]^ = 1 of the area fraction as well as for vanishing affinity contrast Δ*U*^2,1^ = 0. In the following, we will use the term “uni-env membrane” as an abbreviation for “membrane in a uniform environment”.

The phase diagrams in Figures 4–6 below are based on the exact binodals of the lattice binary model as described by (17) and on the exact values of the spectator phase mole fractions *X_a_*_,*_^[1]^ and *X_a_*_,*_^[2]^ as given by (63)–(66) and (56)–(59) for small and large values of the affinity contrast. Therefore, the phase diagrams in Figures 4–6 are exact as well. In contrast, the phase diagrams in Figure 7 are based on the mean-field approximation.

In all phase diagrams displayed in Figures 4 ,5, 6 and 7 below, the two coexistence regions for the membrane segments are distinguished by their color: the coexistence regions of segment $S^{\left[1\right]}$ are blue whereas the coexistence regions of segment $S^{\left[2\right]}$ are red. Furthermore, the remaining white regions in the phase diagrams represent one-phase regions, in which the membranes attain uniform compositions without domains.

### 7.2. Phase Diagrams for Positive Affinity Contrasts

In Figure 4, we display the phase diagrams of adhering membranes in the (*X_a_, T/T_c_*)-plane as obtained from the lattice binary mixture for small and large *positive* values of the affinity contrast Δ*U*^2,1^ = Δ*U*^[2]^ − Δ*U*^[1]^ keeping the area fraction *q*^[1]^ of segment $S^{\left[1\right]}$ constant. The relative affinities Δ*U*^[^*^m^*^]^ are related to the molecular interaction potentials *U_a_*^[^*^m^*^]^ and *U_b_*^[^*^m^*^]^ via Δ*U*^[^*^m^*^]^ = *U_a_*^[^*^m^*^]^ − *U_b_*^[^*^m^*^]^ which implies that

(67) $$U^{2,1}>0\;\;\;\text{iff }\;\;U_{b}^{\left[2\right]}-U_{b}^{\left[1\right]}<U_{a}^{\left[2\right]}-U_{a}^{\left[1\right]}.$$

Because we use the sign convention that attractive interaction potentials are negative, see (32), the second inequality in (67) applies to systems, in which the *b*-molecules prefer to stay in segment $S^{\left[2\right]}$ while the *a*-molecules prefer to stay in segment $S^{\left[1\right]}$. Therefore, as one increases the mole fraction *X_a_* starting from *X_a_* = 0, the *a*-molecules are first enriched in segment $S^{\left[1\right]}$, which then undergoes phase separation leading to the blue coexistence regions in Figure 4.

For *large* positive values of the affinity contrast Δ*U*^2,1^ as shown in Figure 4a, the two coexistence regions differ only in their width but have the same shape. For *small* positive values of *U*^2,1^ as shown in Figure 4b, the two coexistence regions provide a decomposition of the coexistence region of the uni-env membrane, the latter being depicted by the broken line in Figure 4a.

The phase diagrams in Figure 4 correspond to fixed area fraction $q^{\left[1\right]}=A^{\left[1\right]}/A=0.7$. In general, this area fraction can vary in the range 0 ≤ *q*^[1]^ ≤ 1. In Figure 5, we show how the phase diagram evolves with increasing values of *q*^[1]^ for large, positive values of the affinity contrast Δ*U*^2,1^. For *q*^[1]^ = 0, the adhering membrane consists of segment $S^{\left[2\right]}$ only and we are left with the red coexistence region only. For small nonzero values of *q*^[1]^, a narrow blue region appears at small values of *X_a_*, see Figure 5a. As we further increase the area fraction *q*^[1]^ and, thus, the relative size of segment $S^{\left[1\right]}$, the blue region grows at the expense of the red region, see Figure 5b–d. Finally, as we reach the limiting value *q*^[1]^ = 1, the adhering membrane consists only of segment $S^{\left[1\right]}$ and we are left with the blue coexistence region only. Thus, as we change the area fraction *q*^[1]^ for fixed affinity contrast Δ*U*^2,1^, the phase diagram in the (*X_a_, T/T_c_*)-plane evolves smoothly for all values of *q*^[1]^ including the limiting values *q*^[1]^ = 0 and *q*^[1]^ = 1. In the latter cases, we recover the coexistence region for the uni-env membrane as depicted by the broken line in Figure 5.

All phase diagrams shown in Figures 4 and 5 have the same topology: the single coexistence region for the uni-env membrane, *i.e.*, the membrane in a uniform environment, is replaced, for nonzero affinity contrast Δ*U*^2,1^, by two coexistence regions, a blue one for segment $S^{\left[1\right]}$ and a red one for segment $S^{\left[2\right]}$. For *T >* 0, these two coexistence regions are separated by an intermediate one-phase region that lies within the coexistence region of the uni-env membrane. This generic topology of the phase diagrams has interesting consequences for experimental observations. Thus, consider a two-component membrane that is phase separated when exposed to a uniform environment. After this membrane is segmented by adhesion, the phase separation is restricted to one of the two membrane segments, *i.e.*, phase separation can be observed either in segment $S^{\left[1\right]}$ or in segment $S^{\left[2\right]}$ but not in both segments simultaneously. Furthermore, for mole fractions and temperatures that correspond to the intermediate one-phase regions between the two coexistence regions of the membrane segments, the phase separation in the uni-env membrane is suppressed by the adhesion-induced segmentation.

### 7.3. Phase Diagrams for Negative Affinity Contrasts

In Figure 6, we display the phase diagrams of adhering membranes in the (*X_a_, T/T_c_*)-plane as obtained from the lattice model for small and large *negative* values of the affinity contrast Δ*U*^2,1^ = Δ*U*^[2]^ − Δ*U*^[1]^ keeping the area fraction *q*^[1]^ of segment $S^{\left[1\right]}$ constant. When the relative affinities Δ*U*^[^*^m^*^]^ are expressed in terms of the molecular interaction energies *U_a_*^[^*^m^*^]^ and *U_b_*^[^*^m^*^]^, we now find that

(68) $$\Delta U^{2,1}<0\;\;\;\text{iff }\;\;U_{a}^{\left[2\right]}-U_{a}^{\left[1\right]}<U_{b}^{\left[2\right]}-U_{b}^{\left[1\right]}$$

Because we use the sign convention that attractive interaction energies are negative, see (32), the second inequality in (68) applies to systems, in which the *a*-molecules prefer to stay in segment $S^{\left[2\right]}$ while the *b*-molecules prefer to stay in segment $S^{\left[1\right]}$. Therefore, as one increases the mole fraction *X_a_* starting from *X_a_* = 0, the *a*-molecules are first enriched in segment $S^{\left[2\right]}$, which then undergoes phase separation leading to the red coexistence regions in Figure 6.

The phase diagrams for negative values of the affinity contrast are intimately related to those for positive values. This relation can be understood as follows. Instead of changing the sign of the affinity contrast Δ*U*^2,1^, we could also interchange the names of the *a*- and the *b*-molecules. We would then obtain the same phase diagrams as in Figures 4 and 5 but with *X_a_* replaced by *X_b_*. If we now redraw these diagrams in terms of *X_a_* = 1 − *X_b_*, we swap the relative positions of the blue and red coexistence regions and recover the phase diagrams for negative values of the affinity contrast as shown in Figure 6.

### 7.4. Phase Behavior for Variable Affinity Contrasts

The variation of the area fraction *q*^[1]^ for fixed affinity contrast *U*^2,1^ leads to a smooth evolution of the coexistence regions in the (*X_a_, T/T_c_*)-plane as shown in Figure 5. In contrast, the coexistence regions undergo abrupt changes as we vary the affinity contrast Δ*U*^2,1^ from small positive to small negative values, or *vice versa*, for fixed values of the area fraction *q*^[1]^.

As an example, consider the phase diagram for *small negative* affinity contrasts Δ*U*^2,1^ and *q*^[1]^ = 0.7 as shown in Figure 6b. This phase diagram exhibits a relatively narrow red coexistence region for mole fractions *X_a_* in the range 0 ≲ *X_a_* ≲ 0.3 and a relatively broad blue coexistence region for mole fractions within 0.3≲ *X_a_* ≲ 1, separated by a very narrow one-phase region. For *vanishing* affinity contrast Δ*U*^2,1^ = 0, this one-phase region has disappeared and the red and blue regions have merged into the single coexistence region for the uni-env membrane, see broken lines in Figures 4 and 6. Finally, for *small positive* affinity contrasts Δ*U*^2,1^, the blue and the red coexistence regions swap their relative positions: the relatively broad blue coexistence region is now located at 0 ≲ *X_a_* ≲ 0.7 and the relatively narrow red coexistence region at 0.7 ≲ *X_a_* ≲ 1. Thus, as we change the sign of the affinity contrast, the phase diagrams change in an abrupt and discontinuous manner.

The abrupt changes of the (*X_a_, T/T_c_*) phase diagrams arising from variations of the affinity contrast are limited to the vicinity of Δ*U*^2,1^ = 0. Indeed, as long as we do not cross Δ*U*^2,1^ = 0, a continuous change of the affinity contrastΔ*U*^2,1^ leads to a smooth variation of the two coexistence regions within the (*X_a_, T/T_c_*)-plane. This latter property is illustrated in Figure 7, which displays such a smooth variation for negative values of *U*^2,1^ as obtained from the mean-field approximation to the lattice model defined in Section 5.2.

## 8. Summary and Outlook

In this paper, we first emphasized that the adhesion of membranes often leads to two membrane segments, denoted by $S^{\left[1\right]}$ and $S^{\left[2\right]}$, that are in contact with two different environments. Examples are provided by the adhesion of vesicles, see Figure 2, by hole- or pore-spanning membranes, see Figure 3a, and by membranes supported by chemically patterned surfaces, see Figure 3b. We then studied how these adhesion geometries affect the phase behavior of two-component membranes and vesicles.

Our theoretical analysis was based on the configurational energies ℰ^[1]^ and ℰ^[2]^ for the two membrane segments as described by the expression (39), which generalizes the standard lattice model for binary mixtures to the different adhesion geometries considered here. In the configurational energies ℰ^[1]^ and ℰ^[2]^, the interactions of the *a*- and *b*-molecules with the different environments are taken into account by the relative affinities Δ*U*^[1]^ and Δ*U*^[2]^ as defined in (33). These relative affinities shift the relative chemical potential Δ*μ* = *μ_a_* − *μ_b_* for the *a*- and *b*-molecules within the two membrane segments. From these shifts alone, we can conclude that the two membrane segments undergo two distinct phase transitions for Δ*U*^[1]^ ≠ Δ*U*^[2]^ as follows from the relations in (42).

In order to obtain theoretical predictions that are accessible to experiments, we then considered the mole fractions *X_a_*^[1]^ and *X_a_*^[2]^ in the two membrane segments and showed how these mole fractions determine the phase diagrams as a function of the overall mole fraction *X_a_* and the dimenionsless temperature *T̄* via the partitioning relation (44) and the chemical equilibrium between the two membrane segments. As a result, we found that the phase behavior of the adhering membranes depends, in general, on four parameters: overall mole fraction *X_a_*, temperature *T̄*, area fraction *q*^[1]^, and affinity contrast Δ*U*^2,1^ = Δ*U*^[2]^ − Δ*U*^[1]^ as defined in (49).

For the generic case of nonzero affinity contrast, the phase diagrams for the adhering membranes contain two distinct coexistence regions in the (*X_a_, T̄*)-plane separated by an intermediate one-phase region as shown in Figures 4–7. These different regions evolve smoothly as one changes one of the four parameters except for variations of the affinity contrast Δ*U*^2,1^ across the hyperplane defined by Δ*U*^2,1^ = 0. The latter behavior is illustrated by the phase diagrams in Figures 4b and 6b, which correspond to small positive and small negative values of the affinity contrast, respectively.

All phase diagrams shown in Figures 4–7 have the same topology. This universality has interesting consequences for experimental observations. Thus, consider a two-component membrane or vesicle that is phase separated when exposed to a uniform environment. When this membrane or vesicle is brought into contact with two different environments that lead to a nonzero affinity contrast, see the examples in Figure 2 or Figure 3, the phase separation is confined to one of the two membrane segments, *i.e.*, phase separation may be observed either in segment $S^{\left[1\right]}$ or in segment $S^{\left[2\right]}$ but not in both segments simultaneously. Furthermore, if the mole fraction and temperature of the adhering membrane belong to the intermediate one-phase region between the two two-phase coexistence regions in the (*X_a_, T̄*)-plane, phase separation and domain formation are suppressed by the adhesion-induced segmentation. In this way, we predict generic features of the adhesion-induced phase behavior that can be scrutinized by experiment.

The theory described here can be extended and generalized in several ways. First, it is possible to study the dynamics of the phase separation processes, which proceed via the formation and coarsening of intramembrane domains within the two membrane segments, by simulations of the lattice model. We have already performed preliminary Monte Carlo simulations that support the phase diagrams described in this paper. Second, one can apply the theoretical approach used here for the lattice binary mixture to any two-component membrane. One exampe is provided by binary cholesterol/DPPC mixtures, for which the phase diagram in Figure 1 has been deduced [5,8]. This phase diagram is primarily based on nuclear magnetic resonance measurements, which reveal intramembrane domains. In the study presented here, we adopted the view that these domains arise from phase separation into liquid-ordered and liquid-disordered phases in agreement with the latest data analysis [5] and the recent review in [8]. It has also been suggested that the domains may arise via different mechanisms such as enhanced composition fluctuations or the formation of molecular complexes as reviewed in [6]. The experimental confirmation of the adhesion-induced phase behavior described here would provide rather strong evidence for domain formation via phase separation. Third, our theory can be extended to membranes in contact with an arbitrary number of environments as well as to membranes containing three or more molecular components as will be shown elsewhere.

For two-component membranes exposed to two different environments as considered here, the phase diagrams depend on four parameters, three of which are easy to determine experimentally. Indeed, the mole fraction *X_a_* the temperature *T* are standard thermodynamic control parameters while the area fraction *q*^[1]^ can be controlled by the design of the adhesion system. The remaining parameter provided by the affinity contrast Δ*U*^2,1^ could also be determined experimentally. For adhering vesicles, for instance, one can measure the adhesion energy of the bound membrane segment for different membrane compositions, from which the relative affinities of the bound membrane segment and, thus, the affinity contrast can be deduced. Alternatively, one may also obtain these relative affinities from molecular dynamics simulations of atomistically resolved membranes.

## Figures and Tables

**Figure A1:**
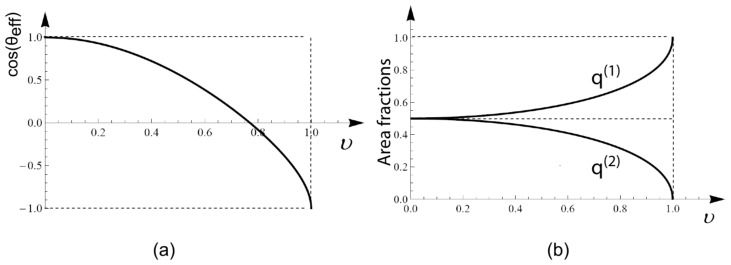
Spherical cap geometry: (**a**) Cosine of the effective contact angle *θ*_eff_ and (**b**) Area fractions $q^{\left[1\right]}=A^{\left[1\right]}/A$ and $q^{\left[2\right]}=A^{\left[2\right]}/A$ of the unbound [1]-segment and the bound [2]-segment as a function of reduced vesicle volume *v*.

## Appendix

### A. Spherical Cap Shape of Adhering Vesicles

In this appendix, we consider the spherical cap shape of an adhering vesicle in the strong adhesion regime as shown in Figure 2 and collect a few simple geometric relations.

First, the area fractions $A^{\left[1\right]}/A$ and $A^{\left[2\right]}/A$ can be expressed in terms of the effective contact angle *θ*_eff_ via

(69) $$q^{\left[1\right]}=\frac{A^{\left[1\right]}}{A}=\frac{2}{3+\text{cos}(\theta_{\text{eff}})}$$

and

(70) $$q^{\left[2\right]}=\frac{A^{\left[2\right]}}{A}=\frac{1+\text{cos}(\theta_{\text{eff}})}{3+\text{cos}(\theta_{\text{eff}})}$$

Likewise, the reduced volume *v* of the vesicle satisfies the relation

(71) $$v=2\frac{{\left[1-\text{cos}(\theta_{\text{eff}})\right]}^{1/2}[2+\text{cos}(\theta_{\text{eff}})]}{{\left[3+\text{cos}(\theta_{\text{eff}})\right]}^{3/2}}$$

Inverting this latter relation numerically, one obtains

(72) $$\text{cos}(\theta_{\text{eff}})=f(v)$$

as shown in Figure A1a. When (72) is inserted into (28), the area ratio *q*^[1]^ assumes the form

(73) $$q^{\left[1\right]}=\frac{A^{\left[1\right]}}{A}=\frac{2}{3+f(v)}=\frac{2}{3+\text{cos}(\theta_{\text{eff}})}$$

for 0 ≤ *v* ≤ 1 as displayed in Figure A1b. For *v* = 0, the vesicle has a pancake shape with contact angle *θ*_eff_ = 0 and area fraction $q^{\left[1\right]}=\frac{1}{2}$, for *v* = 1, it has a spherical shape with *θ*_eff_ = *π* and *q*^[1]^ = 1. The relation (73) shows that the area fractions can be directly determined by measuring the effective contact angle *θ*_eff_.

For constant area A and volume V, the areas $A^{\left[1\right]}$ and $A^{\left[2\right]}$ of the two membrane areas are fixed. On the other hand, if the volume of the adhering vesicle is changed by osmotic inflation or deflation, its contact area will decrease and increase, respectively, as described by relation (73) and shown in Figure A1b. Therefore, the vesicle volume provides an experimentally accessible control parameter that can be used to change the area fraction *q*^[1]^.
